# The Reconstruction of a Bronze Battle Axe and Comparison of Inflicted Damage Injuries Using Neutron Tomography, Manufacturing Modeling, and X-ray Microtomography Data

**DOI:** 10.3390/jimaging6060045

**Published:** 2020-06-08

**Authors:** Maria Mednikova, Irina Saprykina, Sergey Kichanov, Denis Kozlenko

**Affiliations:** 1 Department of Theory and Methods, Institute of Archaeology RAS, 117036 Moscow, Russia; ia.ras@mail.ru; 2 Frank Laboratory of Neutron Physics, Joint Institute for Nuclear Research, 141980 Dubna, Russia; ekich@nf.jinr.ru (S.K.); denk@nf.jinr.ru (D.K.)

**Keywords:** cultural heritage, Bronze Age, 3D model reconstruction, weapon injuries reconstruction, neutron tomography, X-ray microtomography

## Abstract

A massive bronze battle axe from the Abashevo archaeological culture was studied using neutron tomography and manufacturing modeling from production molds. Detailed structural data were acquired to simulate and model possible injuries and wounds caused by this battle axe. We report the results of neutron tomography experiments on the bronze battle axe, as well as manufactured plastic and virtual models of the traumas obtained at different strike angles from this axe. The reconstructed 3D models of the battle axe, plastic imprint model, and real wound and trauma traces on the bones of the ancient peoples of the Abashevo archaeological culture were obtained. Skulls with traces of injuries originate from archaeological excavations of the Pepkino burial mound of the Abashevo culture in the Volga region. The reconstruction and identification of the injuries and type of weapon on the restored skulls were performed. The complementary use of 3D visualization methods allowed us to make some assumptions on the cause of death of the people of the Abashevo culture and possible intra-tribal conflict in this cultural society. The obtained structural and anthropological data can be used to develop new concepts and methods for the archaeology of conflict.

## 1. Introduction

In recent years, more attention from archaeologists and other history-related scientists has been focused on comprehensive studies of the great number of cultural heritage items using natural science methods. The ever-growing number of scientific research methods is being used to study the physical and chemical properties of ancient coins [1,2], metal weapons [3,4], and stone, glass, and ceramic archaeological objects [5]. The obtained results are intended to be used to expand our knowledge about ancient production technologies, the origins of ore mining, the degree of preservation, and corrosion and crack penetration [6,7]. The uniqueness and great value of museum archaeological items require modern, innovative approaches to their study. Besides this, nondestructive structural imaging methods such as neutron and X-ray tomography are advantageous [8,9].

In our work, we want to present not only the capabilities of structural imaging methods [7,8,9,10] but also bioarchaeological-related methods of historical reconstruction of the identification processes of damage caused by metal weapons of the Bronze Age based on human remains. Our joint studies are dictated by the requests of one scientific direction in archaeometry science, namely, conflict archaeology [11,12,13]. There are many archaeological finds connected to the manifestations and determinations of great violence between individual humans and cultural groups in the past [11,13]. The reasons, evolution, and passing of conflicts can be derived from joint research by physicists and anthropologists. Studies of ancient weapons and the remains of peoples can be used to recover the battle opportunities and experiences of the distant past. Modern osteology techniques and methods are used for reconstruction of the wounds, injuries, and traumas of ancient warriors or civilians. In the frame of the historical theory of conflicts, Bronze Age metal battle axes are indicators of a great degree of ancient society militarization [11]. For these joint studies, we chose a bronze axe found on the forest-steppe territory of the area of the Abashevo archaeological culture, dated from the end of the 3rd millennium B.C. This culture is characterized by developed livestock, agricultural farming, and bronze metalworking, in which it has several points of linkage with the Eurasian metallurgical province [14,15]. The axe was excavated in the Malo-Kizilsky settlement (territory of Eastern Abashevo) in the modern Ural region of the Russian Federation. On the other hand, one of the famous archaeological sites of the Abashevo culture is the Pepkino burial mound [16,17] in the Volga region, Russia. This collective burial site contains the remains of 27 persons bearing traces of violent deaths. The nature of the injuries indicates that the enemies used bows in the first wave, and then they attacked with battle axes in close combat. Those Abashevo men from the Pepkino burial who did not fall from arrows immediately, fought hard. Their skulls show many traumas from strike weapons [17]. The archaeology of conflict declares this tragic situation a result of the political and social situations of the tribes of the Abashevo archaeological cultures or their interaction with neighboring cultures. In addition to the remains of bones in the Pepkino burial, utensils, jewelry, bone amulets, and, particularly interestingly, production molds for bronze axes similar to the above-mentioned were found.

In order to expand our knowledge in the archaeology of conflict, the cultural origin of the found bronze axe, the technology of its manufacture, and the relation of this axe to the received lethal wounds on the skulls of the remains of people from the Pepkino burial mound were studied. In our research, modern imaging methods like neutron and X-ray microtomography were utilized. Manufacturing modeling based on the found molds was performed. We believe that the presented bioarchaeological results of our joint research can form a basis for some conclusions in the archaeology of conflict about ancient Abashevo cultural evolution and its cross-cultural interactions.

## 2. Materials and Methods

**Figure 1:**
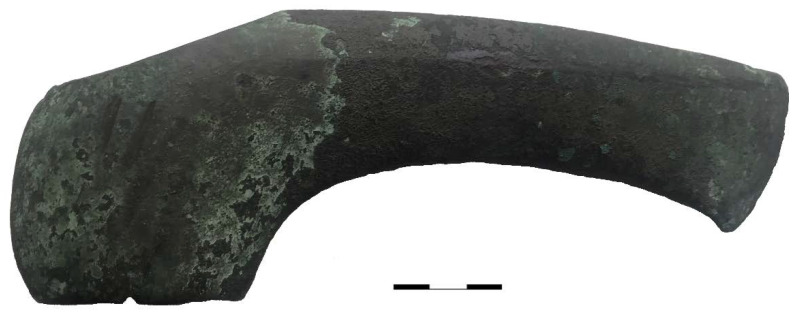
A photo of the bronze axe from the Malo-Kizilsky settlement of the Abashevo culture. A scale line of 30 mm is presented.

The bronze battle axes of the Abashevo archaeological culture are heavy and lethal weapons. For our studies, we selected a real bronze battle axe of the Abashevo culture from the ancient settlement Malo-Kizilsky, Russian Federation [18]. This archaeological site was discovered in 1948 by archaeologist K.V. Salnikov [14]. The settlement consists of several huts, burials, and skeletal remains. It is assumed that this settlement was destroyed and burned down as a result of an external attack [17]. The weight of the studied axe is 953 g. Its blade length is 209 mm, the blade width is 50 mm, and the width of the hitting edge is about 6 mm and 0.3 mm at the sharp part (Figure 1). This axe could inflict deep, lethal head traumas, including penetrating wounds. The bone remains with similar wounds were collected from the Pepkino burial mound in the Volga-Kama archaeological area. Based on visual observations, the majority of the human skulls there have traumatic wounds inflicted by a battle axe [17]. Both archaeological sites (Malo-Kizilsky and Pepkino) belong to the same Abashevo culture, and the finds correspond to approximately the same period. The connection between the shape and structure of the real bronze axe and the inflicted wounds on the bones is the aim of the presented studies.

X-ray microtomography of the bone samples from Pepkino was performed using FEI HELISCAN. X-ray tomography with an accelerating voltage of 20–160 kV can provide single projections, circular or spiral tomography of samples with resolution 0.8 μm, and tomography with a double spiral trajectory, or with an iterative trajectory. FEI HELISCAN provides an opportunity to perform running tomography of samples with length/diameter up to 8 cm, reconstruction of tomography data, and visualization of 2D virtual slices.

Modeling of the bronze axe hitting edge was performed by an imprint of plasticine materials heated up to 110 °C based on the tomography reconstruction model. MicroCT scanning of the obtained plasticine model was performed. Bioarchaeological comparisons of the axe model with the three-dimensional (3D) data of cranial traumas were performed. Thermo Scientific™ PerGeos Software (Systems for Microscopy and Analysis, Moscow, Russia) was used for the segmentation and visualization of virtual data.

The neutron tomography experiments were performed at a neutron radiography and tomography facility [19,20], with the subject placed on beamline 14 of the IBR-2 high-flux pulsed reactor at JINR, Dubna. The characteristic parameter L/D was 200 in the neutron experiments. A set of neutron radiography images was collected by a detector system based on a high-sensitivity camera with a HAMAMATSU S12101 CCD chip. The ^6^LiF/ZnS(Cu) scintillation screen manufactured by RC TRITEC Ltd (Teufen, Switzerland) was used for converting neutron radiation into visible light. The field of view of the detector is 200 mm × 200 mm. The tomography experiments were performed with a rotation step of 0.5°; the total number of measured radiography projections was 360. The exposure time for one projection was 20 s. The imaging data were corrected by the camera dark current image and normalized to the image of the incident neutron beam using ImageJ software [21]. The tomographic reconstruction was performed by utilizing SYRMEP Tomo Project (STP) software [22]. Finally, a data set containing the volume distribution of 3D pixels (voxels) was reconstructed. The size of one voxel in our studies is 52 µm × 52 µm × 52 µm. The 3D volume data of voxels are the essence of the spatial distribution of values of the neutron attenuation coefficients inside the sample volume. Attenuation of the neutron beam corresponds with scattering and absorption losses inside the material [8]. VGStudio MAX 2.2 software from Volume Graphics (Heidelberg, Germany) was used for the visualization and analysis of reconstructed 3D data.

## 3. Results and Discussion

### 3.1. Neutron Tomography of the Bronze Battle Axe

**Figure 2:**
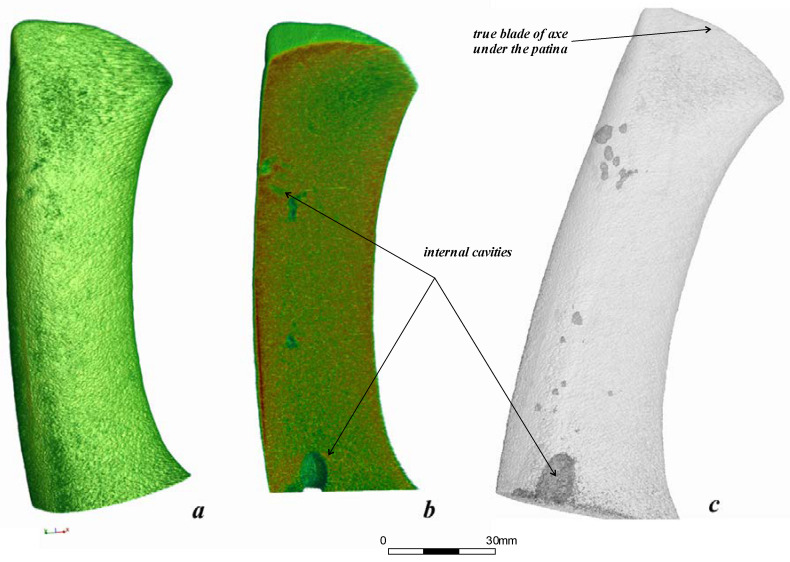
(**a**) 3D model of the bronze axe after tomographic reconstruction from neutron data. (**b**) Longitudinal virtual slice of the 3D model of the bronze battle axe. The inner voids and cavities are visible. The bottom large void is a manufacturing defect. The rainbow-like coloring shows the neutron absorption degree from low (blue) to high (red). (**c**) Transparent virtual model of the bronze battle axe with contrasting internal voids.

The structural model and 3D spatial distribution of internal components of the bronze axe from the Malo-Kizilsky settlement were determined using a neutron tomography method. A set of 360 neutron radiographic images for different angular positions of the metal axe relative to the beam direction was used for 3D tomographic reconstruction of the axe’s inner structure, its casting defects, and hidden traces (Figure 2) [7,8].

The reconstructed 3D model of the bronze battle axe is presented in Figure 2a. The obtained shape of the axe model indicates that it belongs to the sleeve lop-sided type 3 of axes of the Kamsky region [23]. Interestingly, the molds from the Pepkino burial mound correspond to the same type of battle axe.

Neutron absorption inside the axe volume is mostly heterogeneous. Some near-surface regions corresponding to areas of higher neutron attenuation coefficient were detected. We suggest that these are some patina areas [24]. These areas were virtually separated and cut from the total reconstructed volume of the battle axe to improve its model for future calculations. Several hollows and cavities on the surface of the bronze axe are visible (Figure 2b,c). This type of defect most likely corresponds to an effect of insufficient filling of molds or some overheating of the metal during casting. In general, the data obtained indicate that the structure of the internal volume of the axe is quite dense, with no pronounced porosity, except for large casting defects (see below). The volume of the bronze axe consists of 137,774,522 voxels or 19,372.2(1) mm^3^, while the cavity volume forms 1,687,101 voxels or 237.2(1) mm^3^, 1.2(2)% of the total volume.

We obtained the chemical composition of the metal of this axe using an EDAX Orbis PC Micro-XRF Analyzer spectrometer. The studied bronze battle axe from the Malo-Kizilsky settlement is made of arsenic bronze, which is a native alloy of the Tash-Kazgan Deposit with pale ores [23]. This type of bronze alloy is characterized by quality casting and expanded plasticity of copper. As a result, cold forging of arsenic bronzes and a complex slope of the axe dramatically increase the durability of the weapon. However, local arsenic saturation of more than 8% leads to embrittlement of the bronze product. We can assume that this is the reason for the formation of cavities and cracks inside the volume of the studied axe. Also, these cavities can be the result of the release or venting of associated gas during the casting process. This type of defect appears when the melting technology is not strictly enforced: the metal is not deoxidized enough and the molds are not calcined enough.

### 3.2. Bioarchaeological Studies of the Bone Remains Using X-ray Microtomography

Previous bioarchaeological studies of the wounds and injuries on bone remains have sufficient scientific content [12,25,26]. As an experimental example, the artificial skin–skull–brain model was used for the modeling of blunt force traumas from the European Neolithic osteological record [27]. A replica of a Neolithic wooden club was able to produce the wounds in synthetic skulls with remarkable comparisons to Neolithic skeletal remains from an archaeological site in Austria. Fracture formation from blunt force trauma to the skull is dependent on its biomechanical properties. Cranial bone consists of three layers. The outer and inner layers are compact, high-density cortical bone, while the intermediate layer is a low-density, irregularly porous bone structure. Studying fractures, we need to produce lesions on specimens of cranial bone. There are several types of fractures in the case of blunt force trauma, such as linear fractures, produced by a low-velocity force, or depressed fractures with primary, secondary, or even tertiary changes [27]. Speaking of weapon injuries, one should differentiate between blunt force trauma that produces crushing and is indicative of club-like weapons and sharp force trauma that is produced by a bladed weapon. Bioarchaeologists also encounter penetrating wounds from projectiles or pointed weapons. Both linear fractures and comminuted fractures of the cranium are caused by impact with wider objects, whereas depressed and penetrating fractures are a result of narrow objects [28,29] (the last could be a case of bronze battle axe use).

**Figure 3:**
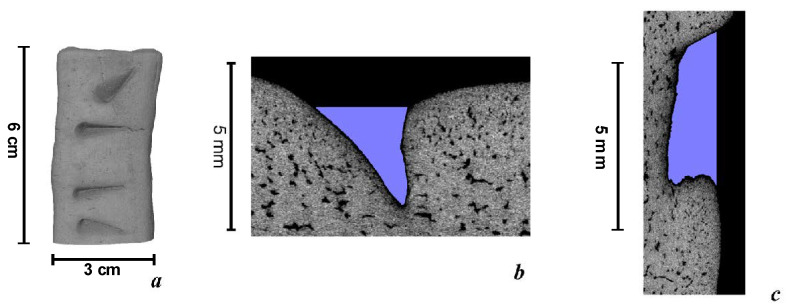
Virtual reconstructed model: (**a**) 3D virtual model of hit tracks in the plasticine obtained from the battle axe from the Malo-Kizylski site, resolution 180; (**b**) virtual transverse slice of the battle axe imprint; (**c**) virtual longitudinal slice of the battle axe imprint, resolution 70 µm.

The ancient molds of the bronze battle axe were found in the Pepkino burial archaeological site. Experimental imprints of several hits by the same battle axe from the Malo-Kysilski site were made using plasticine. The obtained plastic model was converted into a 3D virtual copy using X-ray microtomography methods (Figure 3). The shapes and cutting angles of the hitting edge of the recovered model of the bronze battle axe were used for modeling of the caused traumas on the bones. This model can also be used to identify shallow or superficial wounds that are characteristic of fighting or defending warriors.

**Figure 4:**
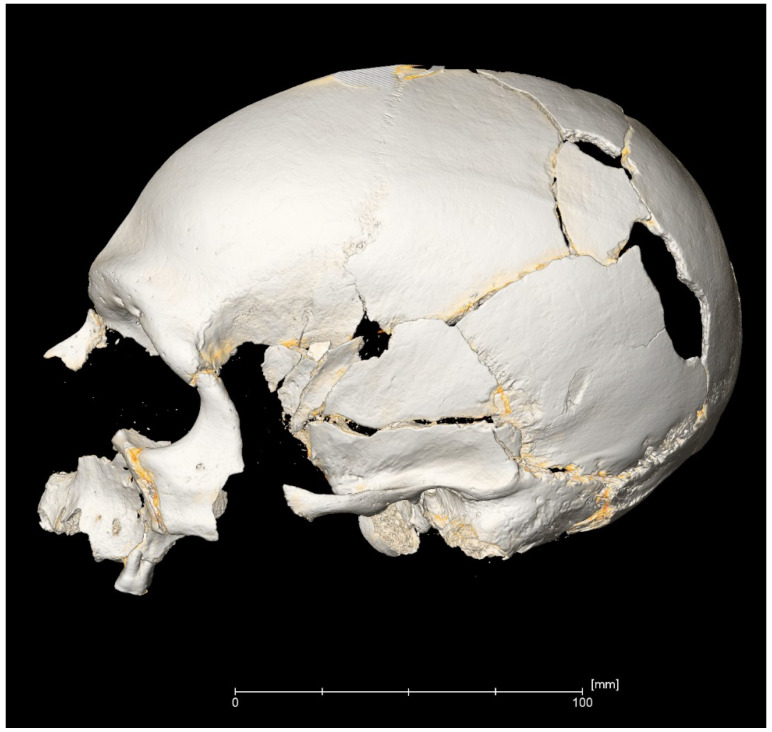
Virtual 3D reconstruction of a male skull from the Pepkino mound with damage from battle axes. The yellow layers are virtual tracks of the beeswax material.

Human skeletal remains from excavations of the Pepkino burial mound bear many traumatic wounds on the skulls and postcranial bones (Figure 4). The primary hypothesis is that young men of the Abashevo culture fell at the hands of enemies, which were the representatives of another tribe or culture [14,16]. After their discovery in the XX century, the skulls of killed people of the Abashevo culture were restored using anthropological paste, including beeswax. The inner and external surfaces of skulls, especially damaged, were covered by these materials. These restored surfaces were tested using X-ray microtomography in order to identify hidden defects. The beeswax layers were virtually separated and removed from the model.

**Figure 5:**
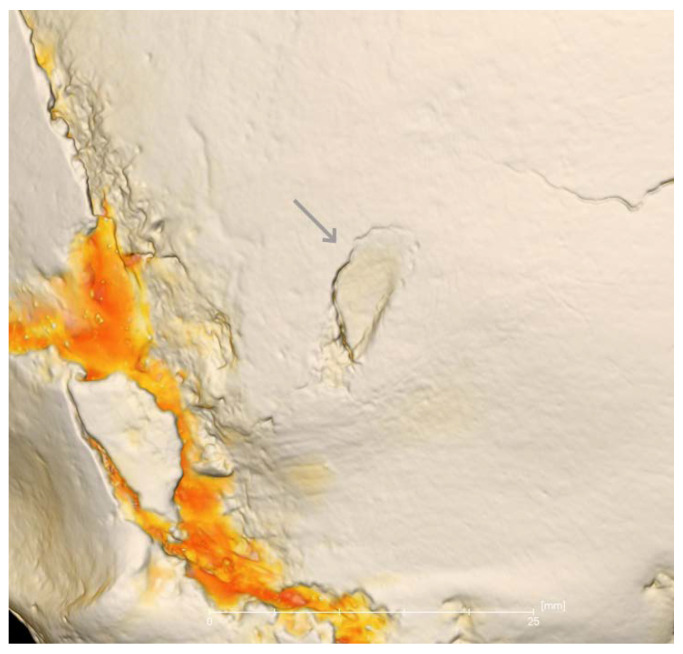
The part of the 3D model of the skull with external damage (arrow) virtually cleaned of restoring beeswax. The yellow layers are the virtual remains of the beeswax material. The resolution is 80 µm.

We identified solid and multiple oval-shaped wounds caused by battle axes. The majority of them, represented by holes surrounded by radiating fractures, were associated with a high-velocity hit by a heavy weapon and could not indicate the type of battle axe directly. However, a superficial oval defect in the lower part of the left parietal bone was visible after X-ray microtomography reconstruction (Figure 5).

**Figure 6:**
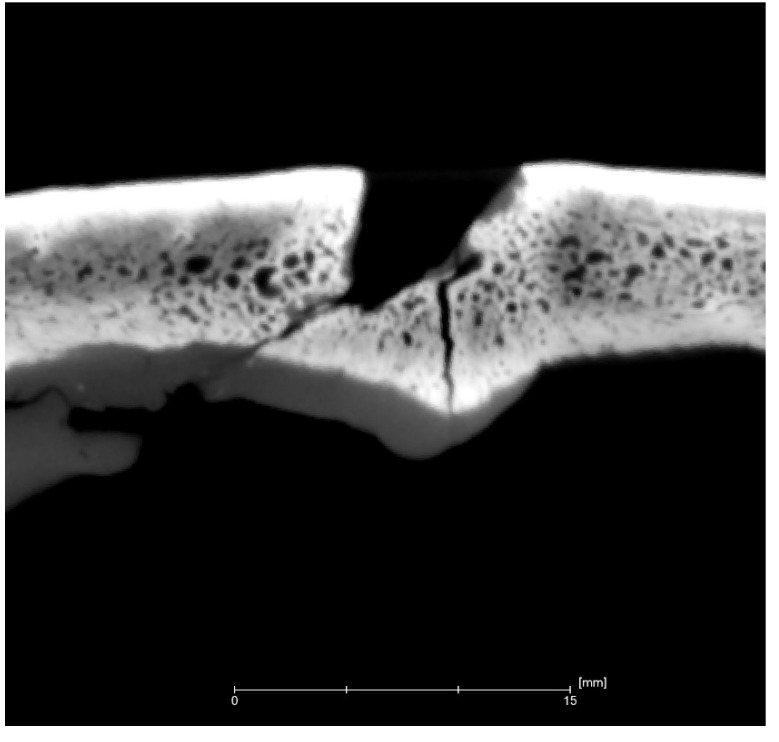
Vertical transverse slice of the cranial vault reflecting the shape of the weapon. The resolution is 190 µm.

A simple explanation for obtaining such injuries is the conclusion that the victim stood face to face with their assaulter and tried to back away from the battle axe, but fell and received other lethal wounds. The superficial trauma by the battle axe as well as serious damage to a bone structure and deep cracks in the skull are visible in the upper part of the model. The virtual slice of the cranial damage reflects the triangle-shaped hitting edge of the bronze battle axe (Figure 6). The shape of the wound looks similar to the shape of the reconstructed plasticine model where the edge hit angle was close to 45° (Figure 3b).

## 4. Conclusions

The modern methods neutron and X-ray microtomography for the 3D visualization and modeling of cultural heritage items were used to reconstruct the model of an ancient bronze battle axe and bone remains with wounds and damage received by people of the Abashevo culture. The production modeling of the battle axe shape from plasticine, together with neutron tomography data, allowed us to identify the bronze axe, determine its type, and obtain bioarchaeological parameters, such as the angles of the blade’s edge and upper hitting part. The obtained 3D data were used to compare injuries and wounds on human skulls with the specifics of the battle axe shape. Microtomography of skulls allowed us to divide the wounds into penetrating and siding wound groups. It is most important that the 3D data of the axe and skulls allowed us to look for conformity of the injuries and the structural models of the battle axes. The comparison of the real bronze axe with the model obtained from molds indicates their complete identity and the belonging of these axes from different archaeological sites of the Abashevo culture to the same cultural group. This conclusion may indicate intra-cultural conflict among the Abashevo people. As a final note, the presented results of quite diverse imaging methods indicate a new direction in the archaeology of conflicts and the applicability of 3D modeling methods to identify both weapons technologies and the specifics of the use of these weapons to injure humans.

## References

[1] Janssens K., van Grieken R. (2005). Non-Destructive Micro Analysis of Cultural Heritage Materials.

[2] Griesser M., Traum R., Vondrovec K., Vontobel P., Lehmann E. (2012). Application of X-Ray and Neutron Tomography to Study Antique Greek Bronze Coins with a High Lead Content. IOP Conf. Ser. Mater. Sci. Eng..

[3] Ling J., Hjärthner-Holdar E., Grandin L., Stos-Gale Z., Kristiansen K., Melheim A.L., Artioli G., Angelini I., Krause R., Canovaro C. (2019). Moving metals IV: Swords, metal sources and trade networks in Bronze Age Europe. J. Archaeol. Sci. Rep..

[4] Salvemini F., Grazzi F., Fedrigo A., Williams A., Civita F., Scherillo A., Vontobel P., Hartmann S., Lehmann E., Zoppi M. (2013). Revealing the secrets of composite helmets of ancient Japanese tradition. Eur. Phys. J. Plus.

[5] Speakman R.J., Glascock M.D. (2007). Acknowledging Fifty Years of Neutron Activation Analyses in Archaeology.

[6] Teixeira J., Magli R., Loupiac C. (2015). Neutron scattering and imaging: a tool for archaeological studies. Eur. J. Miner..

[7] Saprykina I., Kichanov S.E., Kozlenko D.P. (2019). Possibilities, Limitations, and Prospects of Using Neutron Tomography and Radiography for Preservation of Archaeological Heritage Objects. Crystallogr. Rep..

[8] Kardjilov N., Festa G. (2016). Neutron Methods for Archaeology and Cultural Heritage.

[9] Leucci G. (2019). Nondestructive Testing for Archaeology and Cultural Heritage.

[10] Kichanov S.E., Saprykina I., Kozlenko D., Nazarov K., Lukin E., Rutkauskas A., Savenko B. (2018). Studies of Ancient Russian Cultural Objects Using the Neutron Tomography Method. J. Imaging.

[11] Pollard T., Banks I. (2007). War and Sacrifice. Studies in the Archaeology of Conflict.

[12] Redfern R.C. (2017). Injury and Trauma in Bioarchaeology. Interpreting Violence in Past Lives.

[13] Semyan I.A. (2014). Archaeology of Conflicts. On the Problem of Warfare in Sintashta and Petrovka Cultures. Bulletin of the South Ural State University. Ser. Soc. Sci. Humanit. Hist. Sci..

[14] Salnikov K.V. (1954). Abashevskaia kultura na Yuzhnom Urale. (The Abashevo culture in the southern Urals). Sov. Archeol..

[15] Chernykh E.N. (1992). Ancient Metallurgy in the USSR. The Early Metal Age.

[16] Khalikov A.H., Lebedinskaya G.V., Gerasimova V.M. (1966). Pepkinskij Kurgan (Abashevskij Chelovek) (Pepkino Mound (Abashevo Man)).

[17] Mednikova M.B. (2001). Trepanations among Ancient People of Eurasia.

[18] Starikova G.I. (2013). Arheologicheskaya kollekciya Magnitogorskogo istoriko-kraevedcheskogo muzeya (Archaeological Collection of Magnitogorsk Local History Museum). J. Hist. Phililogical Cult. Stud..

[19] Kozlenko D.P., Kichanov S.E., Lukin E.V., Rutkauskas A.V., Bokuchava G.D., Savenko B.N., Pakhnevich A.V., Rozanov A.Y. (2015). Neutron Radiography Facility at IBR-2 High Flux Pulsed Reactor: First Results. Phys. Procedia.

[20] Kozlenko D.P., Kichanov S.E., Lukin E.V., Rutkauskas A.V., Belushkin A.V., Bokuchava G.D., Savenko B.N. (2016). Neutron radiography and tomography facility at IBR-2 reactor. Phys. Part. Nucl. Lett..

[21] Schneider C.A., Rasband W.S., Eliceiri K.W. (2012). NIH Image to ImageJ: 25 years of image analysis. Nat. Methods.

[22] Brun F., Massimi L., Fratini M., Dreossi D., Billè F., Accardo A., Pugliese R., Cedola A. (2017). SYRMEP Tomo Project: a graphical user interface for customizing CT reconstruction workflows. Adv. Struct. Chem. Imaging.

[23] Korenevskyi S.N. (1973). Metallicheskie vtul’chatye topory Ural’skoj gorno-metallurgicheskoj oblasti (Metal lop-sided axes of Ural mining and metallurgical province). Sov. Archaeol..

[24] Kichanov S.E., Nazarov K.M., Kozlenko D.P., Saprykina I.A., Lukin E.V., Savenko B.N. (2017). Analysis of the internal structure of ancient copper coins by neutron tomography. J. Synch. Investig..

[25] Kimmerle E.H., Baraybar J.P. (2008). Skeletal Trauma: Identification of Injuries Resulting from Human Rights Abuse and Armed Conflict.

[26] Shkrum M.J., Ramsay D.A. (2007). Forensic Pathology of Trauma: Common Problems for the Pathologist.

[27] Dyer M., Fibiger L. (2017). Understanding blunt force trauma and violence in Neolithic Europe: The first experiments using a skin-skull-brain model and the Thames Beater. Antiquity.

[28] Knusel C.J., Pearson M.P., Thorpe I.J.N. (2005). The physical evidence of warfare—Subtle stigmata?. Warfare, Violence and Slavery in Prehistory.

[29] Martin D.L., Harrod R.P. (2014). Bioarchaeological Contributions to the Study of Violence. Am. J. Phys. Anthropol..

